# Functional amplification and preservation of human gut microbiota

**DOI:** 10.1080/16512235.2017.1308070

**Published:** 2017-04-10

**Authors:** Nadia Gaci, Prem Prashant Chaudhary, William Tottey, Monique Alric, Jean-François Brugère

**Affiliations:** ^a^ EA-4678 CIDAM, Clermont Université, Université d’Auvergne, Clermont-Ferrand, France

**Keywords:** Fecal microbiota transplantation (FMT), glycerol, dimethylsulfoxide, polyethylene glycol, Environmental Control System for Intestinal Microbiota (ECSIM), cryoprotective agent (CPA), gut microbiota preservation

## Abstract

**Background**: The availability of fresh stool samples is a prerequisite in most gut microbiota functional studies.

**Objective**: Strategies for amplification and long-term gut microbiota preservation from fecal samples would favor sample sharing, help comparisons and reproducibility over time and between laboratories, and improve the safety and ethical issues surrounding fecal microbiota transplantations.

**Design**: Taking advantage of *in vitro* gut-simulating systems, we amplified the microbial repertoire of a fresh fecal sample and assessed the viability and resuscitation of microbes after preservation with some common intracellular and extracellular acting cryoprotective agents (CPAs), alone and in different combinations. Preservation efficiencies were determined after 3 and 6 months and compared with the fresh initial microbiota diversity and metabolic activity, using the chemostat-based Environmental Control System for Intestinal Microbiota (ECSIM) *in vitro* model of the gut environment. Microbial populations were tested for fermentation gas, short-chain fatty acids, and composition of amplified and resuscitated microbiota, encompassing methanogenic archaea.

**Results**: Amplification of the microbial repertoire from a fresh fecal sample was achieved with high fidelity. Dimethylsulfoxide, alone or mixed with other CPAs, showed the best efficiency for functional preservation, and the duration of preservation had little effect.

**Conclusions**: The amplification and resuscitation of fecal microbiota can be performed using specialized *in vitro* gut models. Correct amplification of the initial microbes should ease the sharing of clinical samples and improve the safety of fecal microbiota transplantation.

**Abbreviations**: CDI, *Clostridium difficile* infection; CPA, cryoprotective agent; D, DMSO, dimethylsulfoxide; FMT, fecal microbiota transplantation; G, glycerol; IBD, inflammatory bowel disease; P, PEG-4000, polyethylene glycol 4000 g.mol^−1^; SCFA, short-chain fatty acid; SNR, signal-to-noise ratio

## Introduction

Gut microbes are of prime importance because of the wide variety of roles they perform within the gut, which include helping to shape the host’s gut mucosa, maturation of the immune system, and their contributions at a nutritional level [1,2]. Links are also now well established with various physiological and pathological states, encompassing extraintestinal conditions such as cardiometabolic and cardiovascular diseases, and mental health [3,4]. The biogeography of the gut shows various patterns both longitudinally throughout the gastrointestinal tract and transversally, from the host-human epithelial cells to the lumen [5]. The fecal matter is believed to reflect this microbial diversity and may be considered as a repertoire of microbes associated with the human gastrointestinal tract.

While the preservation of fecal samples for nucleic acid analyses is well established, the functional preservation of individual human fecal microbiota is rarely addressed. Among the important reasons for establishing such methods, this would allow the preservation of samples from large human clinical studies and their sharing among laboratories by reference collection centers. This is also of importance considering fecal microbiota transplantation (FMT), which involves the introduction of filtrated feces from a healthy donor into the gastrointestinal tract of patients as a mean of prevention and therapy. FMT is already used, notably in the case of inflammatory bowel disease (IBD) and *Clostridium difficile* infection (CDI) [6,7], and could be extended to other diseases (e.g. auto-FMT after chemotherapy and the prevention of some chronic diseases).

However, as some gut microbes, most of which are still unknown or known only by a short genomic sequence, are extremely sensitive to oxygen, rapid use of a selected donor’s sample is necessary to avoid the loss of viable microbes over time. This impairs the reproduction of identical inocula over time and limits verification of the potential carriage of pathogens at the time of transplantation, raising ethical and technical problems considering the safety of FMT; these could be overcome by efficient preservation, preceded by amplification of the microbial repertoire. Anaerobic fecal cultures (batch techniques) coupled with −70°C preservation of cultures have been successfully used for CDI [8]. Although this issue was not adressed, it is very likely that this kind of culture method would favor certain microbial populations (those with a short doubling time) to the detriment of others, without permitting the establishment of a microbial balance that would be representative of the microbial ecology of the intestine. Also, most of the available protocols for preservation deal with short-term storage of cultures or peculiar subpopulations of a consortium owing to their biotechnological and pharmacological aspects (e.g. anaerobic ammonium-oxidizing bacteria) [9,10]. Considering the human gut microbiota, some attempts have been made to optimize the storage conditions for fecal samples [11,12] but insufficient information is available. Moreover, assessment of the reliability of preservation techniques should encompass a test to discriminate between living and dead microbial cells. A study published in 2014 used *in vitro* gut-simulating systems to analyze the viability of microbes after preservation [13].

In the present study, microbial cells originating from a fresh stool sample were amplified, then preserved using three cryoprotective agents (CPAs), alone or in combination. We used the *in vitro* gut-simulating Environmental Control System for Intestinal Microbiota (ECSIM) to distinguish between viable and dead cells, and to determine their diversity and metabolic activities. Bacteria and methanogenic archaea from the initial amplification were compared to the initial fecal microbiota, and the preservation efficiencies were determined after 3 and 6 months.

## Materials and methods

### Initial stools sample, media, and culture conditions

Fresh stools were obtained after protocol submission in the Centre Hospitalier Paul Ardier, Issoire, France, with the consent of the donor, an 83-year-old female volunteer with no history of antibiotic treatment. This sample was chosen as it tested positive for all the usual types of methanogens that may be encountered in the human gut [14], i.e. the species *Methanobrevibacter smithii* (*Mb. smithii*), *Methanosphaera stadtmanae* (*Ms. stadtmanae*), and members of the order *Methanomassiliicoccales, Ca*. *Methanomethylophilus alvus* and *Ca*. *Methanomassiliicoccus intestinalis* [15]. The gut environment-simulating system P-ECSIM was used to test the functionality of the microbial preservation, as described previously [16] except that the pH of the media was 6.2. Media used for the generation of inoculum and for the fermentative procedure were the same as described previously [17].

### Initial inoculum generation, preservation of inocula, and regeneration from stored samples

**Figure 1.**
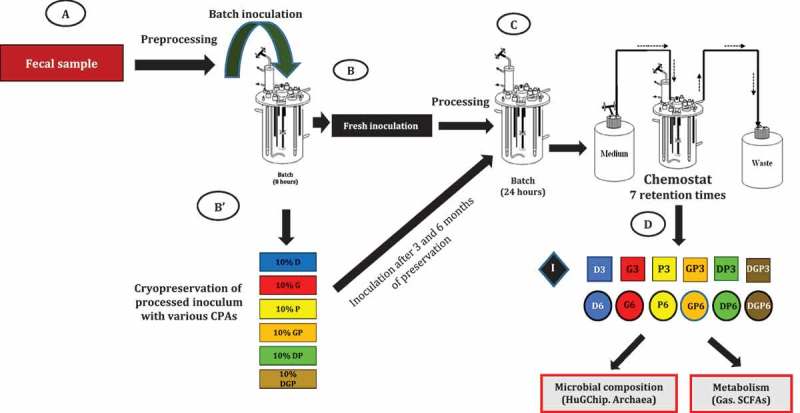
Strategy and experimental design, showing the timeline of fermentation experiments and sampling. A first inoculum is generated from a fresh fecal sample (A), then used directly (B) or after 3–6 months of preservation at −80°C with various cryoprotective agents (CPAs) alone or in combination (B'). During the Environmental Control System for Intestinal Microbiota (ECSIM) experiments (C), samples are taken after 7 retention times of the chemostat process (D). These samples, corresponding to the initial point (black diamond), the 3 month preserved inocula (squares), and the 6 month inocula (circles), are subjected to microbial analyses (HuGChip DNA microarray, quantification of archaea) and to analyses of fermentative gases and short-chain fatty acids (SCFAs). See Materials and Methods for further details.

An overall simplified view of the experimental design is presented in Figure 1. Fermentation was set up by the following steps, performed under anaerobic conditions. First, 1 g of fecal sample (from the 83-year-old female volunteer, hosting both archaea and bacteria) was inoculated in 5 mL of artificial gut medium [15] at 37°C for 10 h immediately after receipt of the sample (collected 4 h before) in an airtight anaerobic box. This preculture was transferred into 95 mL of medium. After 15 h of incubation at 37°C, this culture (~100 mL) was used to inoculate 900 mL of the medium already present in the fermenter and further maintained for 8 h (batch fermentation, pH 6.2, 400 rpm, 37°C). Aliquots of 1 mL with 10% (v/v) glycerol stock, 10% (v/v) polyethylene glycol (PEG), 10% (w/v) dimethylsulfoxide (DMSO), 10% (v/v) glycerol + PEG, 10% (v/v) DMSO + PEG, and 10% (v/v) glycerol + PEG + DMSO were sampled, stored at −20°C for 4 h and then transferred at −80°C. One fresh aliquot was used to inoculate directly the bioreactor for generating the fresh condition that constituted the initial control point.

The stored aliquots were thawed in an anaerobic chamber after 3 and 6 months of storage and inoculated in 5 mL of medium. The same steps of fermenter inoculation were performed as described previously, with the exception that this time fermentation was carried out on a chemostat during seven residence times (7 × 24.96 h) to achieve stabilization [15]. The oxidation/reduction potential (ORP) was monitored using a specific probe. The acquisition and control during the fermentations were handled by C-BIO software (GPC-Global Process Concept). After stabilization, aliquots were sampled for analysis.

### Microbiota composition analysis with HuGChip

Microbial diversity was determined using the HuGChip (GEO: GSE44752), an explorative DNA phylogenetic microarray composed of 3 × 4441 probes targeting 66 bacterial families [18]. DNA was extracted from fermentation samples and the total 16S bacterial gene was amplified using universal primers, labeled with Cy3 or Cy5 dye, and hybridized to the DNA microarray probes as stated previously [18]. After scanning, the data were retrieved using Agilent Feature Extraction software 10.5.1.1 and signals were normalized (Agilent platform GPL16731). The results of the hybridization were deposited in the NCBI database under GEO record GSE77327. Taxonomic assignment of bacterial families was performed as previously described [18], with the help of PhylInterpret software (http://g2im.u-clermont1.fr/HuGChip/), which calculates the relative abundance of bacterial families. When used as a molecular fingerprinting method, probe signals were further normalized between samples to give the same total fluorescence signal. Each probe signal with a signal-to-noise ratio (SNR) above 20 was considered positive.

### Detection and quantification of methanogenic archaea

Methanogenic archaea were assayed by quantitative polymerase chain reaction (qPCR) using the primers described by Mihajlovski et al. [19], targeting *mcrA* (total methanogens and *Mb. smithii*) and *mrtA* (*Ms. stadtmanae*) at annealing temperatures of 58°C, 59°C, and 58°C. The recently discovered *Methanomassiliicoccales* were assayed using the primer pair (5ʹ–3ʹ) GGGGTAGGGGTAAAATCCTG and CGGGGTATCTAATCCCGTTT at 59°C (targeting the 16S rRNA gene). The reactions were performed with the Brilliant III SYBR Green qPCR Master Mix according to the manufacturer’s recommendations using the Mx-3005P apparatus (Agilent Technologies, Santa Clara, CA, USA).

### Gas and short-chain fatty acid analysis

Anaerobic experiments were maintained and tested during the experiment as described previously [17]. Gas chromatography was performed for gas analysis [proportion of carbon dioxide (CO_2_), methane (CH_4_), oxygen (O_2_), and hydrogen (H_2_)] using HP 6890 series columns: Molecular Sieve 5A and Porapack Q (Agilent Technologies, USA) coupled with a flame ionization detector. For short-chain fatty acid (SCFA) analysis, 1 mL of medium was sampled from each fermenter and centrifuged (8000 × *g*, 10 min at 4°C). The supernatant was diluted (1:1) with sterile water and mixed by adding 85 µL of 2-ethylbutyric acid (49 mM) and 20 µL of phosphotungstic acid (500 g.L^−1^). Samples were incubated at 4°C overnight. Samples were then recentrifuged and analyzed for acetate, propionate, butyrate, isobutyrate, isovalerate, valerate, caproate, isocaproate, and heptanoate by gas chromatography (HP 6890 series, column HP-INNOVAX 30 m × 250 μm × 0.25 μm, split ratio = 25:1; Agilent Technologies). 2-Ethyl-butyrate was used as an internal standard. The net production of SCFAs was deduced from the concentration of SCFAs in the reactor [17].

### Statistics

The taxonomic and metabolic profiles were analyzed using the statistical software packages Paleontological Statistics (PAST) [20] and Statistical Analysis of Metagenomic Profiles (STAMP) [21]. For microbiota diversity analysis, Shannon and Simpson diversity indices and other ecological indices were calculated using PAST. Standard statistical tests were performed using MS Excel and GraphPad Prism software.

### Data availability

Data from the HuGChip experiments are available under the GEO record GSE77327 (NCBI database).

## Results

### Experimental design for amplifying microbiota and testing preservation

The overall experimental design is depicted in Figure 1 and detailed in the Materials and Methods section. Fresh stools from a volunteer known to host archaea and bacteria (1 g) were first inoculated in 5 mL of medium mimicking gut substrates [17], and progressive scaling up was then performed up to 1 L with several incubation steps. This allowed any components of the feces that could interfere with experimental interpretations to be diluted as much as possible. Then, 1 mL aliquots were taken, one of which was used directly (see next subsection). The others were mixed with various CPAs under strictly anaerobic conditions, stored first at −20°C for 4 h, followed by 3 and 6 months’ preservation at −80°C. The CPAs used in this study were two cell-permeating, intracellular agents [glycerol (G) and DMSO (D), each at 10% (v/v)] and one extracellular, non-permeating, high molecular weight compound [PEG 4000 g.mol^−1^ (PEG-4000, P) at 10% (w/v)]. They were used alone (D, G, and P) or in combination (DP, GP, and DGP). After 3 and 6 months (3 months and 8 days/6 months and 8 days for DP, GP, and DGP inocula), these aliquots were used to inoculate the ECSIM model simulating the gut’s environmental conditions [15]. Once chemostat conditions had been launched and stabilization reached [after 7 retention times (RT)], several analyses were performed to describe the resulting microbiota, both functionally and structurally.

### Amplification of microbes from fresh starting material

The ECSIM model was inoculated with the 1 mL aliquot that was untreated and not frozen (see above). After 7 RT, several analyses were performed (gas, SCFA, and microbial contents) and compared to the fresh fecal sample (see Supplementary Figure S1 for complete comparisons with initial stools). Data at this stage corresponded to the initial point (T0), and were then used for comparison in identical experiments reproduced 3 or 6 months later using −80°C preserved 1 mL aliquots as the starting inoculum.

### Preservation of metabolic behavior assessed with gas and SCFA production

**Figure 2.**
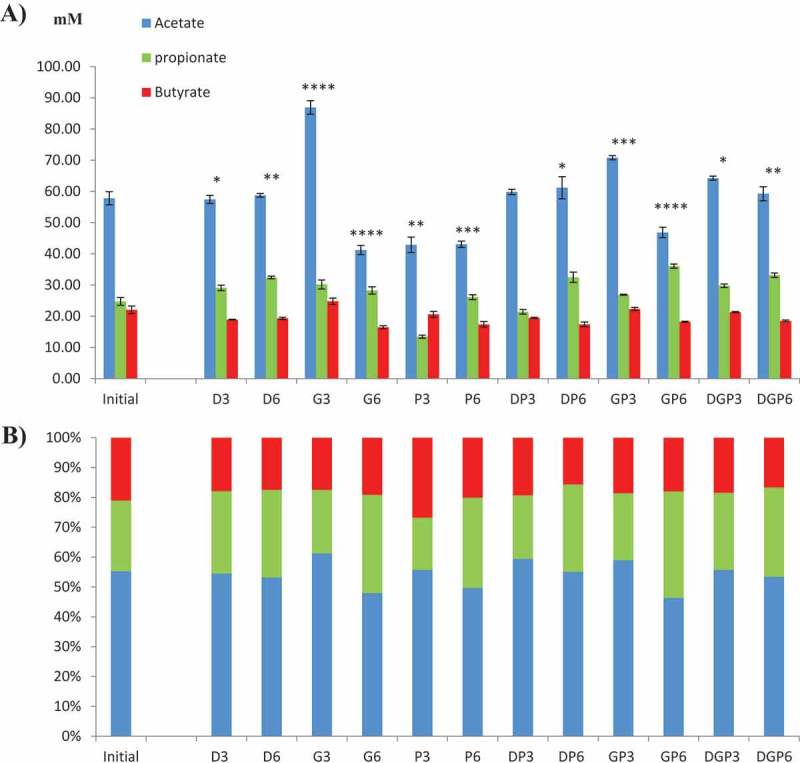
Production of major short-chain fatty acids (SCFAs). (a) Concentration and (b) proportion of the three major SCFAs (acetate, propionate, and butyrate) in the reactors after inoculation with preserved microbiota. Complete results with other minor SCFAs are given in Supplementary Table S2. In (a), the bars indicate SEM. Significance of the effect of treatments and time on the production of the three major SCFAs (two-way analysis of variance, initial vs cryopreservatives and their combinations) is indicated as follows: no indication, not significant; *p ≤ 0.05; **p ≤ 0.01; ***p ≤ 0.001*; ****p ≤ 0.0001.

The ECSIM system allows chemostatic conditions under anaerobia caused by the microbiota fermentative metabolism itself. Similar patterns of gas were obtained for all the preservation conditions (Supplementary Table S1). In brief, H_2_ was detected at low levels (less than 1%), indicating the efficient activity of hydrogenotrophs, as also sustained by the detection of methane. Therefore, all the preservatives used here led to the survival of at least some methanogenic archaea members. Also, the low H_2_ level and the presence of methane are indicative of an efficient fermentation yield: among them, SCFAs are important functional metabolites of the gut microbiota, predominantly formed of acetate, propionate, and butyrate. Both the quantities and proportions of the three major SCFAs varied with treatment and duration of preservation (Figure 2). Compared with the initial values, a 3 month preservation led to an increase in acetate production, and to a lesser extent propionate, when glycerol was used as the CPA either alone or in combination. This effect was lost after 6 months, resulting mainly in lower yields of the three SCFAs compared to the initial values, with little impact on their proportions. The use of PEG-4000 (alone or with glycerol or DMSO) resulted mostly in a decrease in SCFAs, highlighted at 3 months by the propionate proportion (Supplementary Figure S2). Preservation for 6 months resulted in a greater proportion of propionate whichever CPA was used. The CPAs gave the most similar results when mixed together, after either 3 or 6 months of preservation (DGP3 and 6) (Figure 2).

**Figure 3.**
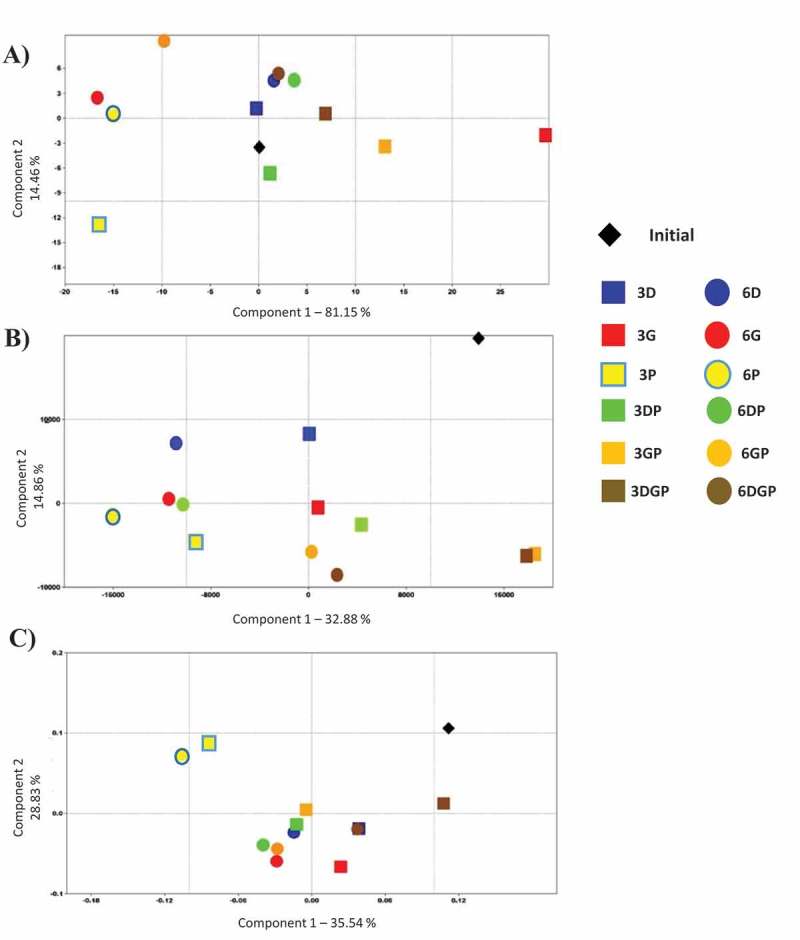
Principal components analysis of microbial composition and metabolism, comparing the samples based on (a) short-chain fatty acid production, (b) HuGChip fingerprint, and (c) taxonomic composition.

Other SCFAs are produced in the human gut, mainly branched SCFAs from the specific metabolism of, notably, proteins and amino acids. Their quantitative analysis showed great discrepancies among the treatments compared to the initial state (Supplementary Table S2). Some (valerate and caproate) were greatly decreased or had even disappeared (heptanoate after 6 months of preservation) with all CPAs, whereas isobutyrate showed an increase in each case. A principal components analysis (PCA) was generated to determine which treatment was the least detrimental to the SCFA profile (Figure 3(a)). This showed that DMSO gave the most similar pattern, considering the first component (contributing to 81.2% of the differences). It was effective either alone or with the other two CPAs, and after 3 or 6 months of cryopreservation. Glycerol and PEG-4000, either alone or together, were the least efficient CPAs. Using PEG-4000 resulted in an important shift but this dissimilar pattern was stable over the two preservation times. It may be concluded from these results that the normobiosis defined at T0 was disrupted by the cryopreservation of inocula.

### High-resolution fingerprint of microbial DNA from resuscitated sources

The (3 ×) 4454 unique probes of the HuGChip DNA microarray [18] were hybridized with full-length 16S DNA amplicons of each microbial DNA retrieved from the fermenters. After normalization, a fingerprint of 570 signals was obtained for the fresh fecal sample. The initial round of fermentation led to a slight increase in the number of positive signals (592, SNR >20), with 562 shared signals. The use of glycerol as the CPA resulted in lower signal numbers, when used alone (around 496 and 514, respectively, compared to 592 after 3 and 6 months of preservation) or together with other CPAs (DMSO and/or PEG-4000), indicating a loss of diversity (Table 1). This decrease was maximal when glycerol was used alone (16.2% after 3 months). The PCA highlights these differences compared with the initial microbiota (Figure 3(b)). The preservation of inocula was best when DMSO and PEG-4000 were used together (Figure 3(b)). These conditions sometimes resulted in a higher number of positive signals, as well as a higher shared signal number with the initial reactor (Table 1). Therefore, it can be deduced that the use of these CPAs gave the best results, but presumably disturbed the initial equilibrium among bacterial populations, resulting in the development of bacteria that were below the detection limit in the initial sample. We consequently questioned which kind of bacteria might be impacted by cryopreservation.

**Table 1.** Number of positive signals on the HuGChip compared with the microbiota obtained after amplification from fresh fecal matter.

Sample	Stool	G3	G6	P3	P6	D3	D6	GP3	GP6	DP3	DP6	DGP3	DGP6
Signals^a^	570	496	514	536	530	579	566	508	515	612	575	552	526
Shared signals^b^	562	420	425	436	432	471	466	424	430	460	448	451	439
Proportion^b^	96.3%	83.8%	86.8%	90.5%	89.5%	97.8%	95.6%	85.8%	87.0%	103.4%	97.1%	93.2%	88.9%

^a^Determined as a cut-off of signal-to-noise ratio above 20.

^b^Compared to the fingerprint of the initial bioreactor (592 positive signals).

### Bacterial composition in resuscitated sources

**Figure 4.**
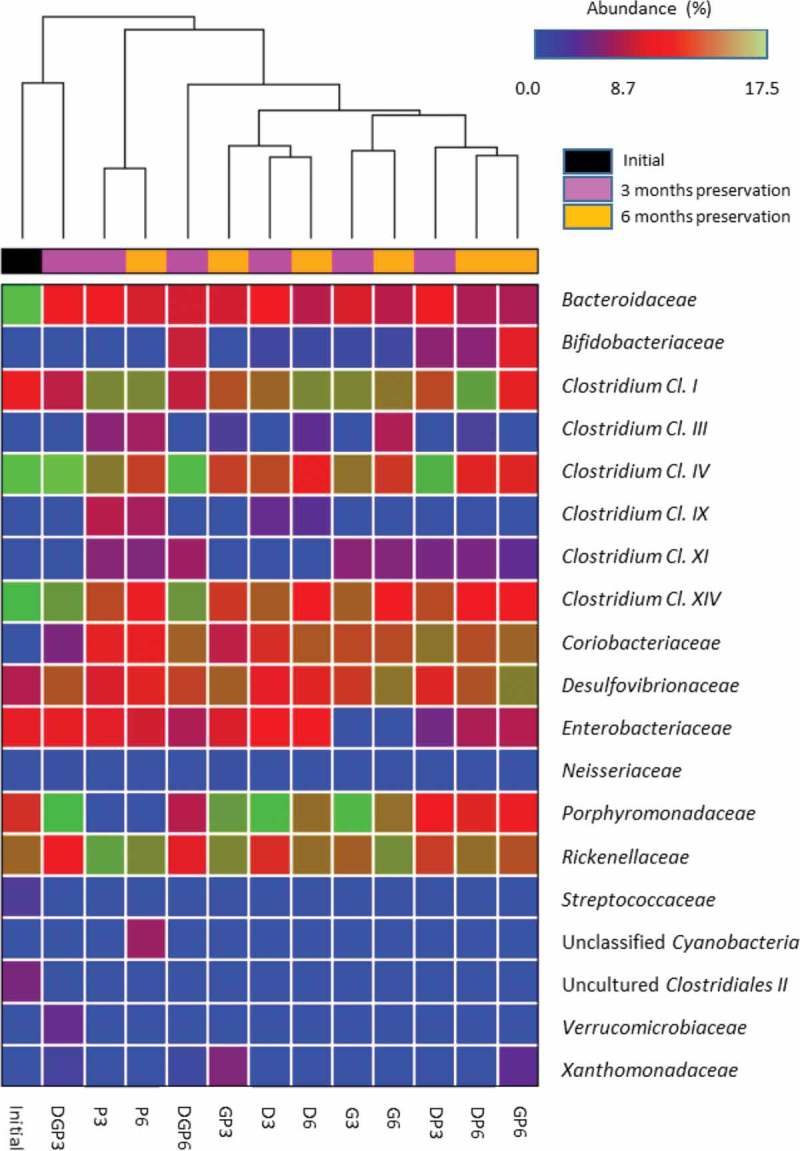
Heat-map comparison of samples based on their microbial composition. The relationship is based on a Ward distance matrix.

The bacterial family proportions of each sample were obtained by complementary analysis of the HuGChip, based on possible 66 bacterial families retrieved from the human gut [18]. The microbiota extracted from fresh fecal matter showed a usual phylum composition of an elderly person’s gut microbiota, with about 50% *Firmicutes*, 30% *Bacteriodetes*, and 20% of *Proteobacteria* (Figure S1). The initial point presented a similar qualitative pattern with a decreased proportion of *Proteobacteria* and an increased proportion of *Firmicutes*. After preservation, *Actinobacteria* were found in every sample, mainly of the family *Coriobacteriaceae* and to a lesser extent of *Bifidobacteriaceae*, which were absent when PEG-4000 was used alone or with DMSO/glycerol (Supplementary Table S3 and Figure S3). Some other bacterial families were recovered belonging to *Firmicutes*, such as *Clostrium* spp. from cluster III (more specifically in longer preservations, except when glycerol and PEG-4000 were used), from cluster IX (present with DMSO and PEG-4000 treatment alone, but not together), and from cluster XI (absent with DMSO alone or with glycerol). Some *Proteobacteria* (families *Xanthomonadaceae* and *Neisseriaceae*) and *Verrucomicrobiaceae* were also revealed when all the CPAs were used together, as well as unknown *Cyanobacteria* (probably those described previously [22,23]) when PEG-4000 was used. In contrast, some bacterial families that were present in the initial sample and the fresh fecal sample disappeared, i.e. *Firmicutes* belonging to uncultured *Clostridiales* of cluster II and *Streptococcaceae*. When analyzing the overall diversity of each sample, higher diversity indices were obtained for the sample preserved with PEG-4000 for 6 months (Table S3). PCA confirms the closer relationship of shorter preservation time (3 months) with the initial point, while only the mixed CPAs DGP were able to sustain a microbiota after 6 months in a similar way to after 3 months (near-confounded points with D3) (Figure 3(c)). Glycerol alone was, however, the worst treatment, compared to all the others either at 3 or 6 months. A heat map was generated using a Ward’s distance matrix to ordinate the samples (Figure 4), which confirmed the close relationship between the initial inoculum and the one preserved with the three CPAs together for 3 months.

### Assessment of the preservation of methanogens

Analysis of stool DNA by qPCR revealed the presence of both the usual *Methanobacteriales* (as well as *Mb. smithii* and *Ms. stadtmanae*) and the far less well known and newly described *Methanomassiliicoccales* (Figure S1). As previously mentioned, CH_4_ in the atmosphere of the fermentation attested to the survival of some of these *Euryarchaea*. qPCR revealed that all three main methanogenic groups (*Mb. smithii*, *Ms. stadtmanae*, and *Methanomassiliicoccales*) were detected from the initial point up to 6 months, whichever CPA was used. The relative proportion of *Mb. smithii* was, however, decreased, resulting in increased proportions of *Ms. stadtmanae* and *Methanomassiliicoccales* (Table S4 and Figure S4).

## Discussion

In this study, we used a continuous fermentation system to first amplify the microbial biomass from the repertoire of bacteria and methanogens from a fresh fecal sample. This process led to a qualitatively similar pattern to the feces when looking at the taxa and SCFAs (Supplementary Figure S1). For example, the HuGChip fingerprint showed 562 shared signals between the fresh feces (570 signals) and the initial amplified microbiota (592 signals) (Table 1). This amplification step has several benefits. First, the amplification seems to preserve the concentration of microbes, as shown in previous studies: the P-ECSIM system gave around 10^9–10^ total anaerobes per milliliter as assessed by microbiological counts versus 10^10^ total anaerobes per gram of fecal matter [16]. Considering also that 1 L is produced by the P-ECSIM, this indicates a 100–1000-fold amplification of microbes. Secondly, the process allows a washing of the initial stools to keep only viable microbes in a defined/controlled medium. This encompasses the dilutions to build the inoculum from fecal matter in addition to the continuous, chemostatic fermentation. Theoretically, considering only this last step, this leads to the renewal of 99.22% of the medium placed at the beginning of the continuous fermentation process. Thirdly, this gives time to analyze the microbiota for specific elements that can be achieved preferably in the latest periods of amplification; in the case of FMT, this encompasses the detection of pathogens (e.g. *C. difficile, Campylobacter, Salmonella, Shigella* spp., and even viruses).

Based on all these advantages, it would be of great interest to preserve the produced microbiota. Preservation of stools for nucleic acid analyses is well documented [24–26] but few methods of preservation of the enrichment cultures, consortia, and co-cultures have been described until now that address the question of amplification and viability of the microbial components together with a way to test their functionality [13]. Amplification and preservation of an initial microbiota would offer great improvements in practice: considering FMT, this would allow the production of tested and reproducible batches of inoculum, while for research purposes, it could authorize the sharing of samples among laboratories, and even the establishment of collection centers devoted to gut microbiota from phenotypically and genotypically characterized hosts. Testing of the functionality and viability of gut microbes in this complex ecosystem after preservation remains challenging, mainly because of the high and still only partly known diversity. Gnotobiotic animals offer one possibility but there are some concerns: besides the inherent difficulties of such models (e.g. cost, infrastructure, and ethical concerns), there are also biases made up of the animal-dependent selection of microbes due to its own immunological, physical, and chemical gut properties [27].

In this study, a way to test the viability of preserved cells from stools is provided by letting microbes grow again and then testing some indicators of the diversity and function of the gut microbiota (i.e. bacterial composition and metabolic behavior). Our survey encompasses one non-bacterial component, the methanogenic archaea, which has been shown to be addressable through *in vitro* systems [28]. They form valuable markers of efficient preservation owing to their high sensitivity to O_2_ and their place in the trophic chain, which reflects the overall functioning of the ecosystem as being hydrogenotrophs [29]. The chemical composition of their cell walls and membranes compared to bacteria [30,31] may also lead to a different response to CPAs. We tested three different CPAs, either alone or mixed, and two different preservation durations, up to 6 months. Methanogenic archaea were successfully preserved by all tested CPAs, encompassing members of the *Methanomassiliicoccales*, for which data in the gut and other environments are still very sparse despite their possible important role in the microbiota ecology and human physiology [29]. Moreover, these three groups are all hydrogenotrophs depending on different methanogenic substrates originating from the gut bacterial metabolism, i.e. CO_2_ (*Mb. smithii*), methanol (*Ms. stadtmanae* and *Methanomassiliicoccales*), and methylamines (*Methanomassiliicoccales*). This also indicates that all of these methanogenic substrates are still generated by specialized bacterial groups, which are therefore preserved by the treatments.

In 2014, a human gut microbiota preservation survey was performed taking advantage of gut-simulating *in vitro* models [13]. Two different treatments were tested, one composed only of DMSO, and the other comprising trehalose and tryptic soy broth plus DMSO. After 3 months at −80°C, the results indicated that both media led to an adequate cryopreservation of fecal matter. In our study, none of the tested CPAs gave a similar pattern compared with the initial point, either structurally or metabolically. This highlights the urgent need for improvements in microbiota preservation processes, which ultimately should be standardized and shared among laboratories and collection centers. Earlier studies demonstrated that DMSO is preferred over other CPAs for the preservation of the microbial consortium [13,32]. Here also, samples with DMSO alone or in combination with other CPAs showed better preservation. This cell-penetrating, permeating agent (as is glycerol) has colligative properties that reduce salt concentration, trigger osmotic effects, and prevent the nucleation and growth of ice crystals that lead to membrane rupture [32]. It has better penetrating power than glycerol, this last also being known to cause a molecular reorganization of plasma membranes during freezing [33]. It has therefore been suggested that glycerol should not be added more than few minutes before freezing [34], a technically difficult requirement when working in an anaerobic chamber, as is needed for fecal microbiota processing. This may explain why glycerol had worse preservation reliability in this study on some checked parameters, even when used in association with other CPAs.

Using non-permeating extracellular agents improves the osmotic imbalance during freezing. PEG-4000 was the only extracellular agent tested in this study. Others, such as trehalose and sucrose, may be used as growth substrates at resuscitation and therefore were not tested but could also be of interest. PEG-4000 showed good performance alone and improved the preservation significantly when used together with permeating agents, probably by better preservation of the cell integrity and by limiting a shortcoming of permeating agents [35]. Importantly, two bacterial families, the *Streptococcaceae* and uncultured *Clostridiales* of cluster II, completely disappeared during the preservation. Some additional CPAs and freezing processes could be tested specifically on isolated members of these families, and then on the whole microbiota, to improve the overall efficiency of the already tested CPAs.

Other improvements are possible. In this study, we preprocessed the sample by a preliminary culture preparation to eliminate non-living fecal components that could interfere with the preservation. We then focused on the cryopreservation of processed samples (not further followed by a lyophilization step) with an easy-to-perform method (4 h at −20°C, followed by −80°C). This step is likely to be a very important one, together with the CPA used, and should be addressed in the future: freezing to −196°C and using liquid nitrogen for conservation can be valuable means by which to increase viability over time. Optimization of the cooling rate could be another important factor. Owing to the extreme diversity of microbes, these steps will have to be determined empirically.

Another important point of this study is that the duration of the preservation (6 months *vs* 3 months) had little effect when DMSO was used (alone or mixed): the HuGChip signals showed a high correlation (*R* ^2^ = 0.965) between the 3 month and 6 month preservation experiments (Supplementary Figure S5). It would be of interest to determine whether preservation at −196°C would improve these results.

## Conclusions

Sustained anaerobia, and a complex and very diverse microbial composition are some of the major properties of the human gut microbiota which lead to difficulties in amplifying and preserving the functionality and viability of the microbiota. Reliable tools to test this viability are also needed. The results obtained in this study provide evidence that *in vitro* systems are a valuable means of fulfilling these objectives, and that permeating and non-permeating CPAs coupled to cryopreservation can be cumulatively used to promote the viability of microbiota.

## Supplementary Material

Supplementary material

## Supplemental material

The supplemental material for this article can be accessed here.
