# Supplementary material for: Functional amplification and preservation of human gut microbiota
Source: Microb Ecol Health Dis. 2017 Apr 10;28(1):1308070. doi: 10.1080/16512235.2017.1308070 (PMC5443092; doi:10.1080/16512235.2017.1308070)
Supplement: Supplementary material [file zmeh_a_1308070_sm8769.zip › 34366-219707-1-SP.pdf]

SUPPLEMENTAL FIGURES

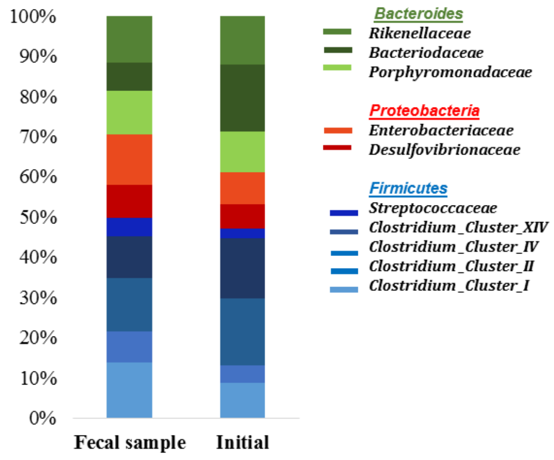

Bacterial composition

	Fecal sample (Copies /µg of DNA)	Initial
Methanomassiliicoccales	37.00 ± $2.56.10^3$	25.6 ± $9.95.10^4$
Mb. smithii	8.94 ± $1.55.10^3$	4.05 ± $5.61.10^4$
Ms. stadtmanae	13.30 ± $3.37.10^2$	50.1 ± $3.62.10^2$

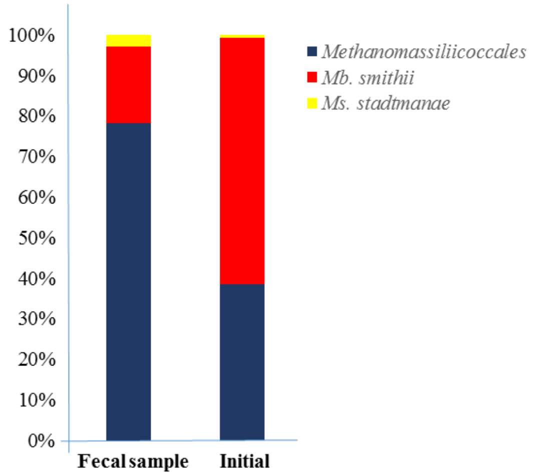

Archaeal composition

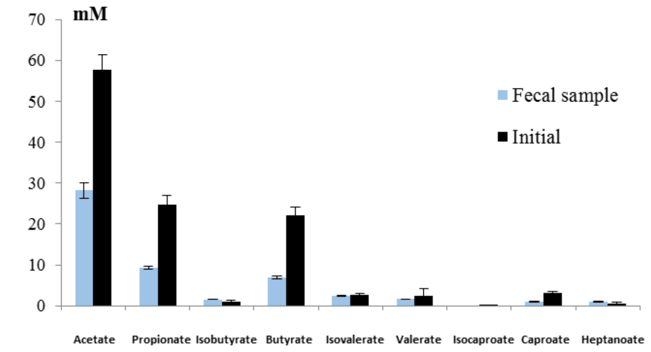

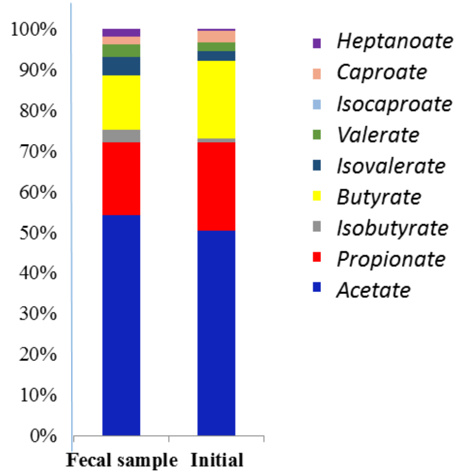

SCFA

Figure S1: Taxonomic and metabolic comparison between fecal microbiota used for this experiment and the microbiota observed after ECSIM simulation with fresh inoculum (reference point of the experiment).

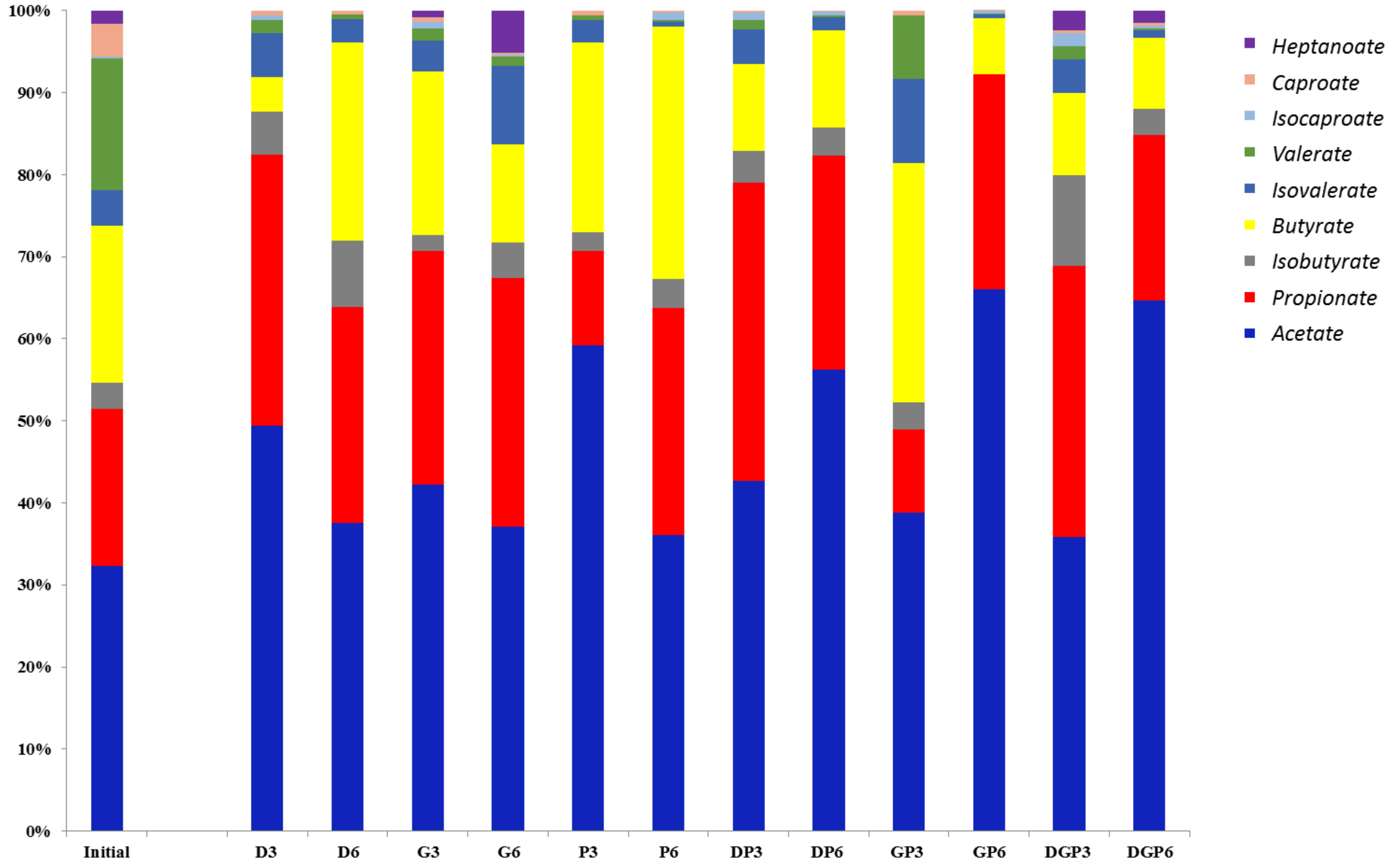

Figure S2: SCFAs proportion.

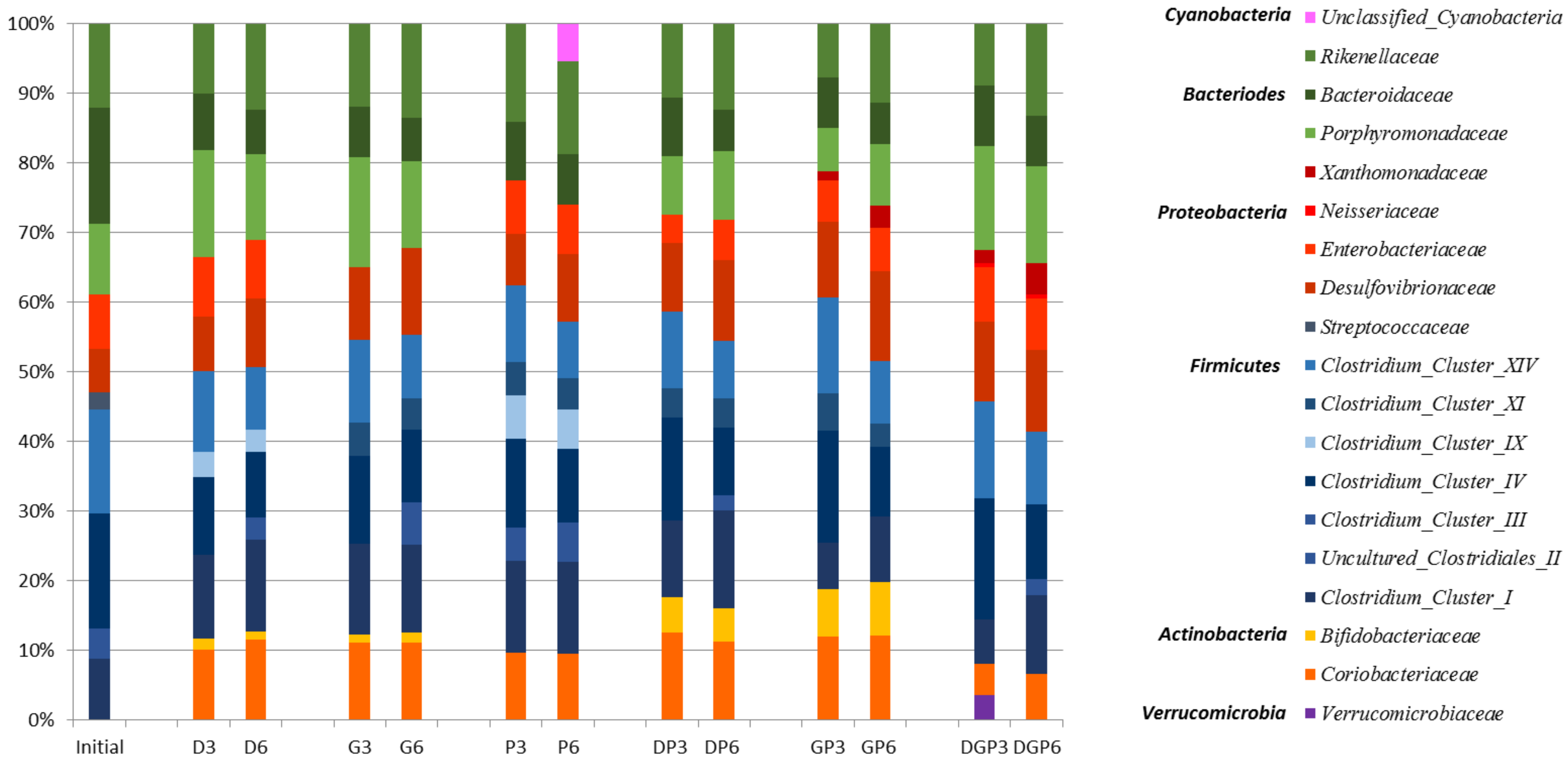

Figure S3: Taxonomic composition of microbiota: proportion of bacteria at the phylum and family level.

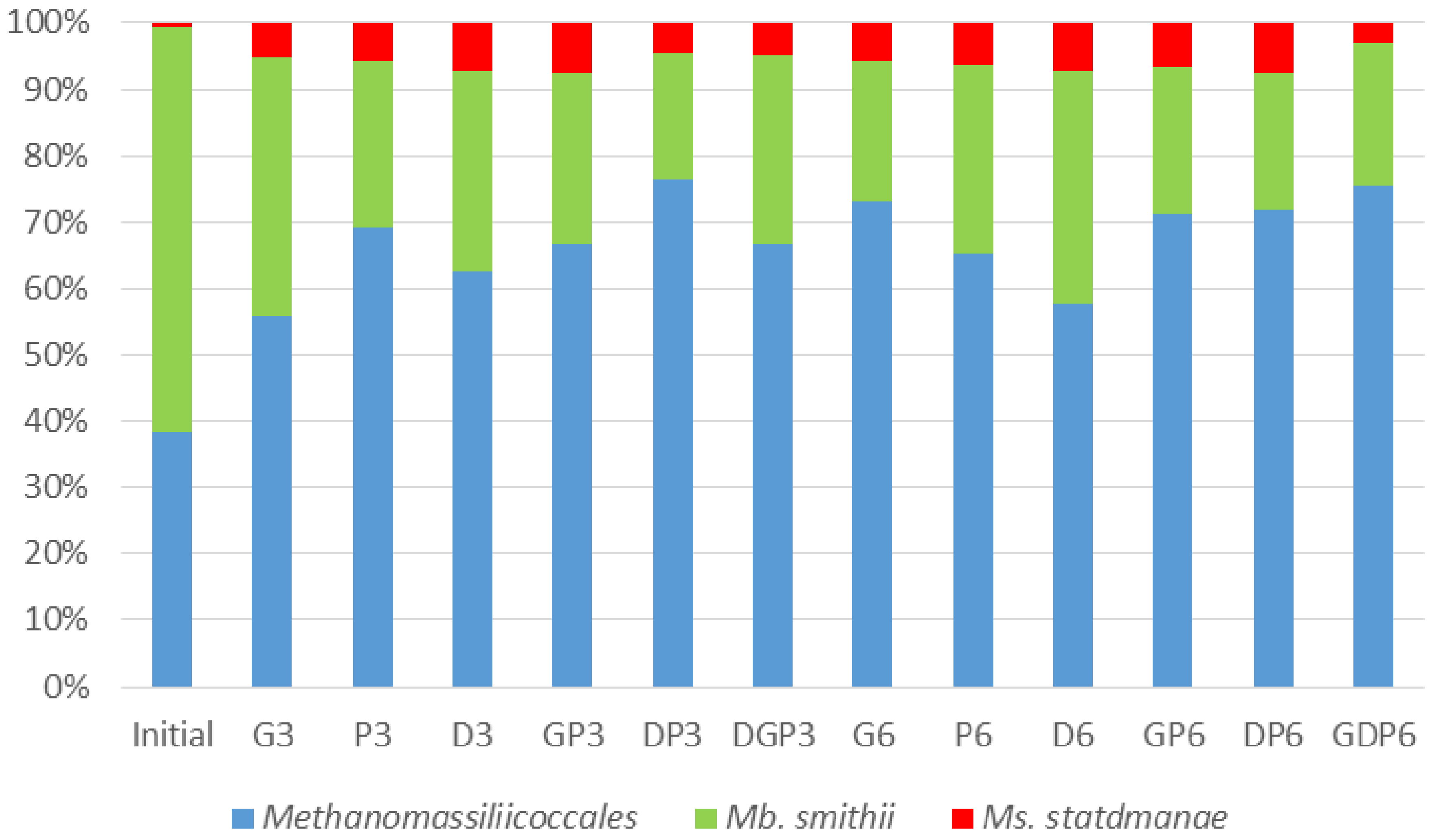

Figure S4: Proportion of methanogenic components (*Methanomassiliicoccales*, *Mb. smithii* and *Ms. stadtmanae*).
