# Supplementary material for: Functional amplification and preservation of human gut microbiota
Source: Microb Ecol Health Dis. 2017 Apr 10;28(1):1308070. doi: 10.1080/16512235.2017.1308070 (PMC5443092; doi:10.1080/16512235.2017.1308070)
Supplement: Supplementary material [file zmeh_a_1308070_sm8769.zip › 34366-219706-1-SP.pdf]

ADDITIONAL TABLES

Table S1: Gas proportion (in %) in the ECSIM atmosphere after completion of seven retention times

	CH_4	CO_2	H_2	O_2	N_2 and others
Initial	7.08 ± 0.15	41.25 ± 0.86	0.12 ± 0.03	0.00 ± 0.00	51.54 ± 0.73
D3	4.83 ± 0.81	49.62 ± 0.60	0.32 ± 0.05	0.00 ± 0.00	45.21 ± 0.56
D6	3.90 ± 0.19	51.20 ± 0.61	0.38 ± 0.06	0.01 ± 0.01	44.49 ± 0.86
G3	3.20 ± 0.22	45.62 ± 2.18	0.36 ± 0.06	0.00 ± 0.00	50.80 ± 2.45
G6	1.23 ± 0.27	52.76 ± 3.60	0.70 ± 0.08	0.00 ± 0.00	45.29 ± 3.44
P3	2.92 ± 0.32	51.64 ± 0.98	0.35 ± 0.16	0.00 ± 0.00	45.07 ± 0.54
P6	1.97 ± 0.28	53.22 ± 1.00	0.48 ± 0.04	0.00 ± 0.00	44.31 ± 0.73
DP3	1.50 ± 0.44	48.53 ± 2.77	0.44 ± 0.43	0.00 ± 0.00	49.52 ± 2.70
DP6	1.60 ± 0.35	49.10 ± 1.43	0.90 ± 0.09	0.00 ± 0.00	48.38 ± 1.67
GP3	2.49 ± 0.21	48.55 ± 2.52	0.54 ± 0.10	0.00 ± 0.00	48.41 ± 2.79
GP6	2.49 ± 0.34	44.46 ± 2.67	0.65 ± 0.08	0.00 ± 0.00	52.39 ± 2.26
DGP3	3.26 ± 0.50	49.18 ± 6.11	0.28 ± 0.02	0.00 ± 0.00	47.26 ± 6.23
DGP6	4.74 ± 0.63	46.55 ± 1.80	0.61 ± 0.02	0.00 ± 0.00	48.08 ± 2.29

Table S2: SCFAs production

(mM)	Acetate	Propionate	Isobutyrate	Butyrate	Isovalerate	Valerate	Isocaproate	Caproate	Heptanoate
**Initial**	57.79 ± 3.63	24.75 ± 2.14	0.91 ± 0.36	22.04 ± 2.15	2.60 ± 0.49	2.45 ± 1.81	0.13 ± 0.02	3.16 ± 0.45	0.63 ± 0.18
**D3**	57.43 ± 2.25	29.11 ± 1.51	3.48 ± 0.24	18.92 ± 0.20	4.94 ± 0.24	0.56 ± 0.07	0.30 ± 0.03	0.14 ± 0.02	0.00 ± 0.00
**D6**	58.73 ± 1.02	32.43 ± 0.72	2.15 ± 0.22	19.32 ± 0.66	2.43 ± 0.08	0.32 ± 0.01	0.00 ± 0.00	0.15 ± 0.01	0.00 ± 0.00
**G3**	86.91 ± 3.75	30.19 ± 2.52	2.91 ± 0.17	24.80 ± 1.77	4.76 ± 0.33	1.01 ± 0.13	0.49 ± 0.08	0.22 ± 0.05	0.27 ± 0.07
**G6**	41.2 ± 2.49	28.25 ± 2.03	5.42 ± 0.29	16.47 ± 0.81	5.22 ± 0.64	0.48 ± 0.08	0.36 ± 0.02	0.14 ± 0.01	0.32 ± 0.34
**P3**	42.88 ± 4.29	13.45 ± 0.84	4.28 ± 0.16	20.62 ± 1.68	6.10 ± 0.20	0.62 ± 0.04	0.00 ± 0.00	0.18 ± 0.05	0.00 ± 0.00
**P6**	43.06 ± 1.78	26.10 ± 1.36	3.61 ± 0.17	17.41 ± 1.51	3.53 ± 0.03	0.32 ± 0.01	0.16 ± 0.05	0.14 ± 0.01	0.00 ± 0.00
**DP3**	59.85 ± 1.48	21.41 ± 1.26	3.88 ± 0.13	19.47 ± 0.37	4.20 ± 0.15	2.36 ± 0.04	0.22 ± 0.03	0.23 ± 0.01	0.00 ± 0.00
**DP6**	61.18 ± 6.13	32.48 ± 2.84	3.15 ± 0.37	17.40 ± 1.30	3.24 ± 0.17	0.32 ± 0.03	0.46 ± 0.05	0.17 ± 0.02	0.00 ± 0.00
**GP3**	70.79 ± 1.14	26.89 ± 0.30	4.28 ± 0.10	22.33 ± 0.86	6.26 ± 0.30	2.66 ± 0.23	0.00 ± 0.00	0.25 ± 0.02	0.00 ± 0.00
**GP6**	46.84 ± 2.39	36.07 ± 0.95	3.58 ± 0.00	18.24 ± 0.25	3.61 ± 0.02	0.46 ± 0.00	0.44 ± 0.01	0.15 ± 0.00	0.00 ± 0.00
**DGP3**	64.24 ± 1.10	29.76 ± 1.02	2.89 ± 0.34	21.31 ± 0.31	4.78 ± 0.13	1.97 ± 0.05	0.15 ± 0.05	0.22 ± 0.01	0.32 ± 0.07
**DGP6**	59.24 ± 3.92	33.15 ± 1.22	2.49 ± 0.19	18.51 ± 0.53	2.05 ± 0.05	0.31 ± 0.01	0.19 ± 0.02	0.22 ± 0.02	0.31 ± 0.09

Table S3: Proportion of bacteria at the family level and diversity indexes

Family	Initial	D3	D6	G3	G6	P3	P6	DP3	DP6	GP3	GP6	DGP3	DGP6
Verrucomicrobiaceae	0,00	0,00	0,00	0,00	0,00	0,00	0,00	0,00	0,00	0,00	0,00	0,04	0,00
Coriobacteriaceae	0,00	0,10	0,12	0,11	0,11	0,10	0,09	0,13	0,11	0,12	0,12	0,04	0,07
Bifidobacteriaceae	0,00	0,02	0,01	0,01	0,01	0,00	0,00	0,05	0,05	0,07	0,08	0,00	0,00
Clostridium Cluster_I	0,09	0,12	0,13	0,13	0,13	0,13	0,13	0,11	0,14	0,07	0,10	0,06	0,11
Uncultured Clostridiales II	0,04	0,00	0,00	0,00	0,00	0,00	0,00	0,00	0,00	0,00	0,00	0,00	0,00
Clostridium Cluster_III	0,00	0,00	0,03	0,00	0,06	0,05	0,06	0,00	0,02	0,00	0,00	0,00	0,02
Clostridium Cluster_IV	0,17	0,11	0,09	0,13	0,10	0,13	0,11	0,15	0,10	0,16	0,10	0,17	0,11
Clostridium Cluster_IX	0,00	0,04	0,03	0,00	0,00	0,06	0,06	0,00	0,00	0,00	0,00	0,00	0,00
Clostridium Cluster_XI	0,00	0,00	0,00	0,05	0,05	0,05	0,04	0,04	0,04	0,05	0,03	0,00	0,00
Clostridium Cluster_XIV	0,15	0,12	0,09	0,12	0,09	0,11	0,08	0,11	0,08	0,14	0,09	0,14	0,10
Streptococcaceae	0,02	0,00	0,00	0,00	0,00	0,00	0,00	0,00	0,00	0,00	0,00	0,00	0,00
Desulfovibrionaceae	0,06	0,08	0,10	0,10	0,13	0,07	0,10	0,10	0,11	0,11	0,13	0,11	0,12
Enterobacteriaceae	0,08	0,08	0,08	0,00	0,00	0,08	0,07	0,04	0,06	0,06	0,06	0,08	0,07
Neisseriaceae	0,00	0,00	0,00	0,00	0,00	0,00	0,00	0,00	0,00	0,00	0,00	0,00	0,01
Xanthomonadaceae	0,00	0,00	0,00	0,00	0,00	0,00	0,00	0,00	0,00	0,01	0,03	0,02	0,05
Porphyromonadaceae	0,10	0,15	0,12	0,16	0,12	0,00	0,00	0,08	0,10	0,06	0,09	0,15	0,14
Bacteroidaceae	0,17	0,08	0,06	0,07	0,06	0,08	0,07	0,08	0,06	0,07	0,06	0,09	0,07
Rikenellaceae	0,12	0,10	0,12	0,12	0,13	0,14	0,13	0,11	0,12	0,08	0,11	0,09	0,13
Unclassified_Cyanobacteria	0,00	0,00	0,00	0,00	0,00	0,00	0,05	0,00	0,00	0,00	0,00	0,00	0,00
**Total**	1,00	1,00	1,00	1,00	1,00	1,00	1,00	1,00	1,00	1,00	1,00	1,00	1,00

	Initial	D3	D6	G3	G6	P3	P6	DP3	DP6	GP3	GP6	DGP3	DGP6
**Simpson_1-D**	0,8774	0,8941	0,8986	0,8829	0,8934	0,898	0,9068	0,8969	0,9015	0,8984	0,9057	0,8854	0,8965
**Shannon_H**	2,18	2,299	2,353	2,193	2,293	2,336	2,427	2,326	2,385	2,369	2,413	2,273	2,335

Table S4: Quantitative PCR results for methanogenic archaea.

	Methanomassiliicoccales		*Mb. smithii*		*Ms. stadtmanae*	
	Copies/µg DNA	SD	Copies/µg DNA	SD	Copies/µg DNA	SD
Initial	2.56E+05	9.95E+04	4.05E+05	5.61E+04	5.01E+03	3.62E+02
G3	5.43E+04	8.15E+03	3.80E+04	4.58E+03	5.11E+03	8.41E+02
P3	7.03E+04	1.61E+04	2.56E+04	1.13E+03	5.71E+03	5.86E+02
D3	7.44E+04	1.02E+04	3.58E+04	1.02E+03	8.74E+03	7.11E+02
GP3	4.56E+04	1.15E+04	1.75E+04	2.41E+03	5.14E+03	5.14E+03
DP3	8.59E+04	6.72E+03	2.14E+04	9.35E+02	5.02E+03	5.34E+02
DGP3	6.00E+04	3.18E+03	2.53E+04	1.02E+03	4.41E+03	1.07E+03
G6	8.31E+04	1.33E+03	2.42E+04	3.62E+03	6.39E+03	1.25E+03
P6	6.25E+04	4.79E+03	2.72E+04	4.89E+03	5.98E+03	6.91E+02
D6	5.87E+04	6.10E+03	3.57E+04	5.41E+03	7.43E+03	2.88E+02
GP6	6.68E+04	7.20E+03	2.09E+04	2.68E+03	6.15E+03	6.73E+02
DP6	8.05E+04	1.37E+04	2.31E+04	7.43E+03	8.60E+03	3.79E+02
GDP6	1.07E+05	9.70E+03	3.02E+04	1.91E+03	4.24E+03	1.41E+03
